# Supplementary material for: Closed-loop auditory stimulation method to modulate sleep slow waves and motor learning performance in rats
Source: eLife. 2021 Oct 6;10:e68043. doi: 10.7554/eLife.68043 (PMC8530509; doi:10.7554/eLife.68043)
Supplement: Transparent reporting form [file elife-68043-transrepform1.pdf]

Westbrook Centre
Milton Road
Cambridge CB4 1YG, UK

P 01223 855340
W elifesciences.org
T @elife

eLife's transparent reporting form

We encourage authors to provide detailed information *within their submission* to facilitate the interpretation and replication of experiments. Authors can upload supporting documentation to indicate the use of appropriate reporting guidelines for health-related research (see EQUATOR Network), life science research (see the BioSharing Information Resource), or the ARRIVE guidelines for reporting work involving animal research. Where applicable, authors should refer to any relevant reporting standards documents in this form.

If you have any questions, please consult our Journal Policies and/or contact us: editorial@elifesciences.org.

Sample-size estimation

- You should state whether an appropriate sample size was computed when the study was being designed
- You should state the statistical method of sample size computation and any required assumptions
- If no explicit power analysis was used, you should describe how you decided what sample (replicate) size (number) to use

Please outline where this information can be found within the submission (e.g., sections or figure legends), or explain why this information doesn't apply to your submission:

- For three experimental groups of closed-loop auditory stimulation:

  1. targeting up-phase,
  2. mock stimulation (no sound),
  3. targeting down-phase,

we expected a standard deviation of 15% for the following group means, referent to delta activity change from baseline (cf. *Ngo et al., 2013; Papalambros et al., 2017; Fattinger et al., 2017*):

1. up-phase = +15%
2. mock = 0%
3. down-phase = -10%

to which we obtained a **n = 8** (for sigma level = 0.05 and power = 0.80).

```
library(daewr)
groupmeans <- c(-10, 0, 15)
> p <- power.anova.test(groups = length(groupmeans), between.var = var(gro
upmeans), within.var = 225, power=0.8,sig.level=0.05,n=NULL)
> p

Balanced one-way analysis of variance power calculation
          groups = 3
               n = 7.93483
     between.var = 158.3333
```

eLife Sciences Publications, Ltd is a limited liability non-profit non-stock corporation incorporated in the State of Delaware, USA, with company number 5030732, and is registered in the UK with company number FC030576 and branch number BR015634 at the address Westbrook Centre, Milton Road, Cambridge CB4 1YG.

```
    within.var = 225
     sig.level = 0.05
         power = 0.8
     NOTE: n is number in each group
```

- This information can be found in the Statistics section of Materials and Methods.

Replicates

- You should report how often each experiment was performed
- You should include a definition of biological versus technical replication
- The data obtained should be provided and sufficient information should be provided to indicate the number of independent biological and/or technical replicates
- If you encountered any outliers, you should describe how these were handled
- Criteria for exclusion/inclusion of data should be clearly stated
- High-throughput sequence data should be uploaded before submission, with a private link for reviewers provided (these are available from both GEO and ArrayExpress)

Please outline where this information can be found within the submission (e.g., sections or figure legends), or explain why this information does not apply to your submission:

- The experimental timeline can be found in the Materials and Methods.
- We recorded EEG/EMG signal for 16 days and tested all animals in single pellet reaching task every day, during the first hour of the light period. All animals went through this procedure once.
- Regional outliers in the EEG/EMG signal were discarded when values were ± 8 IQR from the mean of a 2.5-minute moving window.
- We tested possible outliers before plotting the data by using the Grubbs' test (https://www.graphpad.com/quickcalcs/grubbs1/).
- 1 animal under mock stimulation was excluded from EEG/EMG analysis due to bad signal quality, but kept in the behavioural analysis, since it was part of the group receiving no stimulation.
- The number of animals included in each assessment is stated in each figure legend.

eLife Sciences Publications, Ltd is a limited liability non-profit non-stock corporation incorporated in the State of Delaware, USA, with company number 5030732, and is registered in the UK with company number FC030576 and branch number BR015634 at the address Westbrook Centre, Milton Road, Cambridge CB4 1YG.

Statistical reporting

- Statistical analysis methods should be described and justified
- Raw data should be presented in figures whenever informative to do so (typically when N per group is less than 10)
- For each experiment, you should identify the statistical tests used, exact values of N, definitions of center, methods of multiple test correction, and dispersion and precision measures (e.g., mean, median, SD, SEM, confidence intervals; and, for the major substantive results, a measure of effect size (e.g., Pearson's r, Cohen's d)
- Report exact p-values wherever possible alongside the summary statistics and 95% confidence intervals. These should be reported for all key questions and not only when the p-value is less than 0.05.

Please outline where this information can be found within the submission (e.g., sections or figure legends), or explain why this information doesn't apply to your submission:

- Statistical details can be found in the Materials and Methods, in detail in each section of the Results and, at times, briefly in each figure legend.
- Not all figure legends contain statistical information, due to size constrains.
- Each figure is accompanied by an excel file including all the numerical data and statistical tests applied to each assessment.

(For large datasets, or papers with a very large number of statistical tests, you may upload a single table file with tests, Ns, etc., with reference to sections in the manuscript.)

Group allocation

- Indicate how samples were allocated into experimental groups (in the case of clinical studies, please specify allocation to treatment method); if randomization was used, please also state if restricted randomization was applied
- Indicate if masking was used during group allocation, data collection and/or data analysis

Please outline where this information can be found within the submission (e.g., sections or figure legends), or explain why this information doesn't apply to your submission:

- Animals were randomly distributed into the groups.
- For data analysis, we used the same script (Matlab and R) for all assessments in all animals at once.

Additional data files ("source data")

eLife Sciences Publications, Ltd is a limited liability non-profit non-stock corporation incorporated in the State of Delaware, USA, with company number 5030732, and is registered in the UK with company number FC030576 and branch number BR015634 at the address Westbrook Centre, Milton Road, Cambridge CB4 1YG.

- We encourage you to upload relevant additional data files, such as numerical data that are represented as a graph in a figure, or as a summary table
- Where provided, these should be in the most useful format, and they can be uploaded as "Source data" files linked to a main figure or table
- Include model definition files including the full list of parameters used
- Include code used for data analysis (e.g., R, MatLab)
- Avoid stating that data files are "available upon request"

Please indicate the figures or tables for which source data files have been provided:

- All figures are accompanied by an excel file containing numerical data represented in each plot and statistical information.

eLife Sciences Publications, Ltd is a limited liability non-profit non-stock corporation incorporated in the State of Delaware, USA, with company number 5030732, and is registered in the UK with company number FC030576 and branch number BR015634 at the address Westbrook Centre, Milton Road, Cambridge CB4 1YG.
